# Beyond rings and rods: In-situ imaging reveals the role of VIPP1 in chloroplast homeostasis in tobacco

**DOI:** 10.1093/plphys/kiaf170

**Published:** 2025-05-02

**Authors:** Jitesh Kumar

**Affiliations:** Assistant Features Editor, Plant Physiology, American Society of Plant Biologists; Department of Plant and Microbial Biology, University of Minnesota, Saint Paul, MN 55108, USA; Center for Precision Plant Genomics, University of Minnesota, Saint Paul, MN 55108, USA

Photosynthetic organisms have evolved with the unique ability to convert light energy into chemical energy via photosynthesis. In plants, the chloroplast is the site of photosynthesis, which can be distinguished by a thylakoid membrane (TM) system, that houses necessary light harvesting pigments and reaction centers to initiate the process of photosynthesis. The architecture of the TM has evolved from simpler structures in cyanobacteria to more complex ones in algae and higher plants. In vascular plants like *Arabidopsis* and tobacco, the TM is structured into grana, which are stacks of disc-shaped thylakoids, linked by stroma lamellae (Ostermeier et al. 2024). This organization plays a key role in maximizing light absorption and facilitating the efficient conversion of light energy during photosynthesis. Despite significant research on TM organization, the underlying molecular mechanisms of TM biogenesis and homeostasis in vascular plants remain only partly understood. This includes the trafficking mechanisms for proteins, lipids, and chromophores between the inner envelope or plasma membranes and the TM.

The structure of TM is dynamic and responds to light intensity by undergoing structural changes. Among the key proteins involved in TM remodeling, Vesicle Inducing Protein in Plastid 1 (VIPP1) is essential for TM remodeling, biogenesis, and stress protection in both unicellular organisms and *Arabidopsis* (Gao et al. 2006; Armbruster et al. 2013). In unicellular organisms like cyanobacteria and algae, VIPP1 forms diverse higher-ordered structures, such as ring, helical, stacked rings, and rod-shaped assemblies (Gupta et al. 2021). In contrast, *Arabidopsis thaliana* VIPP1 (AtVIPP1) mostly forms spherical particles rather than rings in vitro. VIPP1 has been shown to strengthen the plant's ability to handle heat stress when overexpressed, while its loss causes lethal phenotype in *Arabidopsis* (Zhang et al. 2012, 2016). However, detailed structural insights were previously lacking.

In this issue of *Plant Physiology*, Gachie et al. (2025) focused on the lack of detailed structural information regarding AtVIPP1 in vascular plants. To address this knowledge gap, authors initiated a comprehensive survey of *PspA/VIPP1* homologues across the plant kingdom. They identified 72 homologues of *PspA/VIPP1* across 41 plant species, with the copy number varying significantly from 1 to 6. For example, *Arabidopsi*s possesses a single *VIPP1* gene, while tobacco contains 2 *VIPP1* homologues (Figure A).

**Figure.**
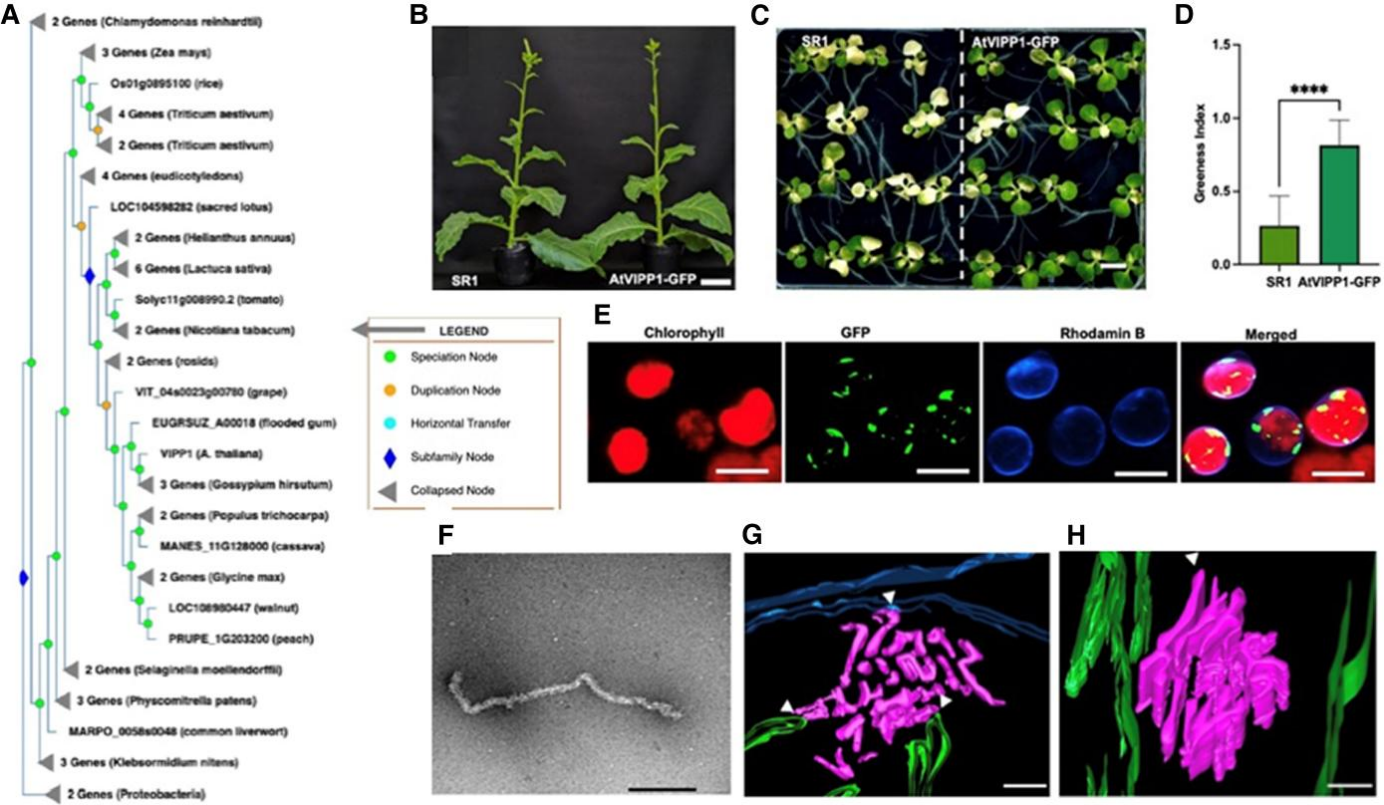
**A)** Phylogenetic tree of VIPP1 from 41 taxonomic groups. **B)** Representative image of wild type (SR1) and transplastomic tobacco line expressing AtVIPP1-GFP. **C** and **D)** Comparison of phenotype and greeness index between SR1 and AtVIPP1-GFP lines post heat stress. **E)** Confocal imaging of purified chloroplasts from transplastomic tobacco line expressing AtVIPP1-GFP. Scale bar = 5 *μ*m. **F)** TEM image showing negatively stained AtVIPP1-GFP oligomers visualized as filament after density gradient centrifugation from tobacco transcriptomic line. Scale bar = 200 nm. **G** and **H)** 3-Dimensional models of AtVIPP1-GFP localization in the chloroplast. Green represents the TM, blue represents the chloroplast envelope, and magenta represents AtVIPP1-GFP structures. Images adopted from Gachie et al. 2025.

To visualize the functional VIPP1 particles (FVPs), the authors created a *Nicotiana tabacum* (tobacco) transplastomic line expressing AtVIPP1-GFP. Using Western-blot analysis, they confirmed high expression of AtVIPP1-GFP compared with the native NtVIPP1. Phenotypic analysis showed no significant differences in growth or photosynthetic activity compared with wild type (SR1) (Figure B). Additionally, the AtVIPP1-GFP expressing line exhibited improved heat stress tolerance, similar to *Arabidopsis* (Figure C and D) (Zhang et al. 2016). Confocal microscopy unveiled large variable AtVIPP1-GFP complexes inside the chloroplasts (Figure E). Interestingly, under heat stress (45 °C), these complexes dynamically reorganized into different shapes, forming transient filament-like structures, suggesting a role in membrane stress response and chloroplast membrane reorganization.

The authors next applied sucrose density gradient centrifugation to purify solubilized tobacco chloroplasts and noted that AtVIPP1-GFP was in the heaviest fraction, suggesting an oligomeric nature. Transmission electron microscopy (TEM) of these fractions revealed filament-like structures (Figure F), like VIPP1 in c*yanobacteria* and *Chlamydomonas*. Further, correlative light and electron microscopy with field emission scanning electron microscop detected GFP fluorescence in thin sections (6 serial sections of 100 nm each), supporting the presence of AtVIPP1-GFP oligomers. TEM analysis showed unique in situ structures, including clusters of electron-dense spheres and bundled filaments. Similar oligomeric structures were observed in a complemented *vipp1-ko Arabidopsis* line, suggesting these could represent functional VIPP1 particles.

To elucidate the 3-dimensional organization of VIPP1 oligomers, the authors employed electron tomography. This technique revealed that AtVIPP1-GFP forms bundled filament-like structures with a diameter of approximately 20 nm. Interestingly, some oligomers were seen associating with the chloroplast inner envelope and the TM, pointing to a possible function in membrane interaction (Figure G). These AtVIPP1-GFP oligomers were also found within the chloroplast envelope and TM at sites of membrane curvature, where they may help stabilize these highly dynamic regions. Furthermore, the presence of oligomers between stacked thylakoid grana hints at their involvement in TM dynamics (Figure H).

This study offers the first comprehensive analysis of the phylogeny, structural characteristics, and the membrane associations of VIPP1 in vascular plants. The authors suggest the variation in VIPP1 structure across species indicates potential functional diversification within the VIPP1 family, similar to ESCRT-III protein family (Carlton and Baum 2023). However, they conclude that VIPP1 gene multiplication does not directly correlate with angiosperm evolution. The observed assemblies of AtVIPP1 oligomers were distinct from those visualized in unicellular organisms, suggesting an evolution related to the complex TM organization in vascular plants. This study uncovered the dynamic nature of FVPs, which respond to heat and osmotic (hypotonic) stress, indicating a protective role in membrane remodeling. The authors also observed protruded thin filaments on FVPs, hinting at potential membrane repair activities through sealing or lipid supply under stress.

The authors were unable to detect FVPs in SR1, unlike in the AtVIPP1-GFP tobacco line, likely due to the sensitivity to chemical fixation. They suggested the C-terminal GFP fusion might have stabilized the FVPs formation as observed in *Arabidopsis vipp1-ko* line complemented with AtVIPP1 without the GFP tag. The FVPs observed in this study resembled *Chlamydomonas reinhardii* VIPP1-mCherry and were located along the TM and plasma membranes. Additionally, the absence of sheet-like structures in tobacco as observed in *Synechocystis*VIPP1 and CrVIPP1 suggest that the GFP tag might hinder their formation, though previous studies indicated this had little effect on FVP formation (Zhang et al. 2016).

This study provides novel insights into the AtVIPP1 assemblies, and understanding the dynamic behavior in response to environmental cues would be exciting to explore. It will be interesting to see whether cryogenic electron tomography may allow the visualization of VIPP1 oligomers in their native form. Since earlier studies have demonstrated interactions of VIPP1 or homologues with proteins like HSP70 and ATPase (Gao et al. 2015; Junglas et al. 2025), it will be intriguing to know the role of such interacting partners in the process of TM homeostasis and TM biogenesis.

## Data Availability

No new data were generated or analysed in support of this research.
